# Supplementary material for: MicroRNA-499 serves as a sensitizer for lung cancer cells to radiotherapy by inhibition of CK2α-mediated phosphorylation of p65
Source: Mol Ther Oncolytics. 2021 Apr 3;21:171–82. doi: 10.1016/j.omto.2021.03.016 (PMC8099482; doi:10.1016/j.omto.2021.03.016)
Supplement: Document S1. Figures S1–S3 and Table S2 [file mmc1.pdf]

OMTO, Volume *21*

Supplemental information

MicroRNA-499 serves as a sensitizer for lung cancer cells to radiotherapy by inhibition of CK2α-mediated phosphorylation of p65

Yu-Shui Ma, Bo-Wen Shi, Hai-Min Lu, Peng-Fei Xie, Rui Xin, Zhi-Jun Wu, Yi Shi, Yu-Zhen Yin, Li-Kun Hou, Cheng-You Jia, Wei Wu, Zhong-Wei Lv, Fei Yu, Gao-Ren Wang, Ji-Bin Liu, Geng-Xi Jiang, and Da Fu

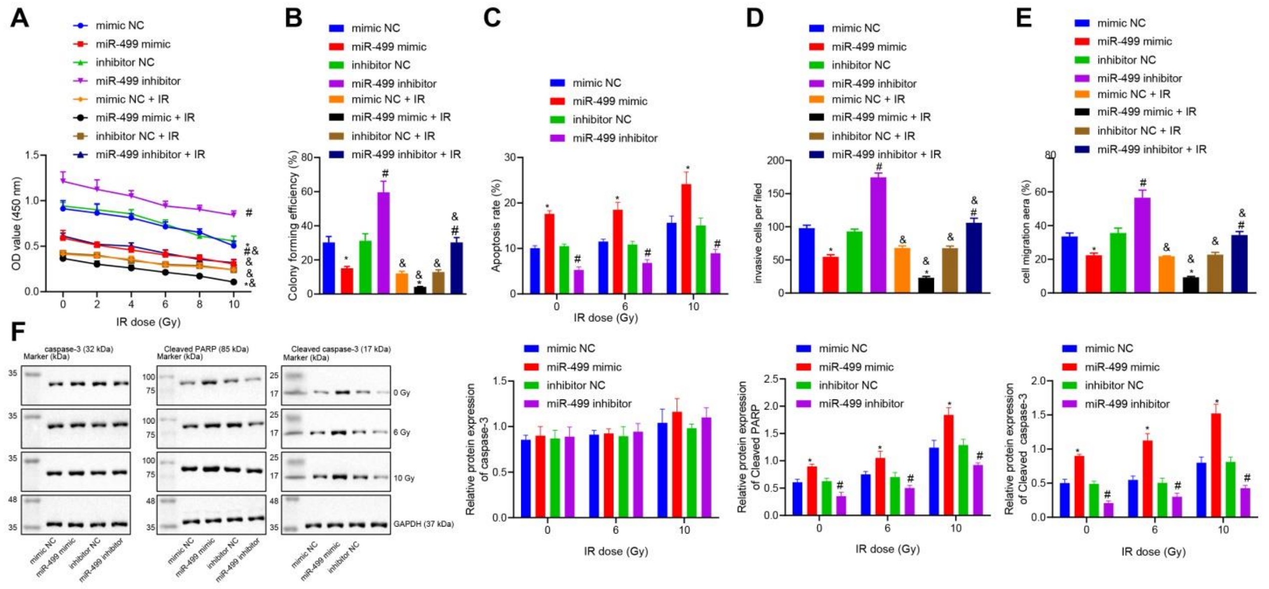

Supplementary Figure 1. Overexpression of miR-499 increased the sensitivity of lung cancer NCI-H292 cells to irradiation (IR) and promoted IR-induced apoptosis *in vitro.* A, Sensitivity of lung cancer cells NCI-H292 to IR detected by MTT assay after overexpression or silencing of miR-499. B, Colonies as measured by colony formation assay in NCI-H292 cells overexpressing or silencing miR-499. * $p < 0.05$ *vs.* NCI-H292 cells treated with mimic NC; # $p < 0.05$ *vs.* NCI-H292 cells transfected with inhibitor NC. C, Apoptosis of NCI-H292 cells as evaluated by flow cytometry after overexpression or silencing of miR-499. D, Invasion assay was performed to examine the effect of overexpression or interference of miR-499 on irradiation-induced invasion of lung cancer cells NCI-H292. E, Scratch assay was performed to examine the effect of miR-499 mimic or miR-499 inhibitor on the migration ability of lung cancer cells NCI-H292 induced by irradiation. F, The protein expression of Caspase-3, Cleaved PARP and Cleaved Caspase-3 measured by Western blot analysis in NCI-H292 cells overexpressing or silencing miR-499. * $p < 0.05$ *vs.* NCI-H292 cells treated with mimic NC; # $p < 0.05$ *vs.* NCI-H292 cells transfected with inhibitor NC. & $p < 0.05$ *vs.* the group with no irradiation. n = 3. The measurement data were expressed as mean ± standard deviation. Data between two groups were compared using independent sample *t*-test. Comparisons among multiple groups were conducted by ANOVA, followed by Bonferroni's *post-hoc* test.

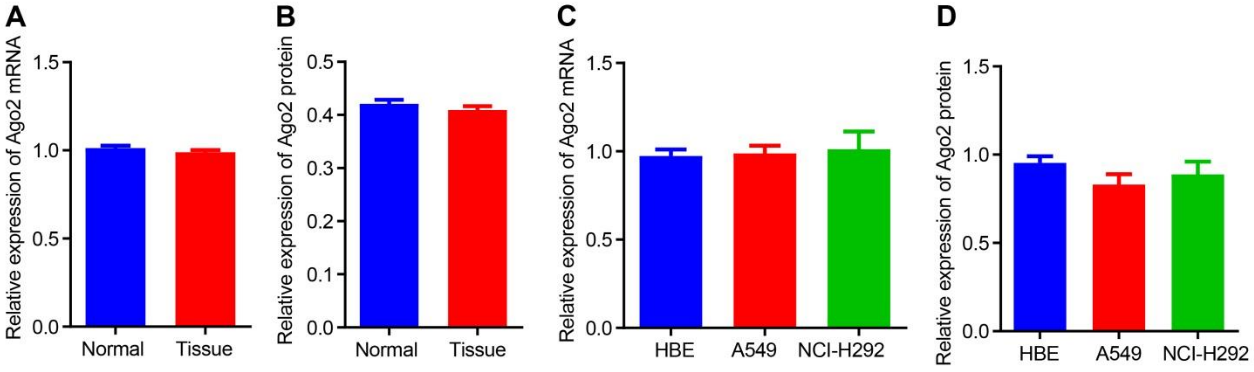

Supplementary Figure 2. A, RT-qPCR was used to detect the difference of Ago2 expression levels between cancer tissues and adjacent normal tissues. B, Western blot was used to detect the difference of Ago2 expression levels between cancer tissues and adjacent normal tissues. C, RT-qPCR was used to detect the difference of Ago2 expression levels between lung cancer cells and immortalized HBE cells. D, Western blot was used to detect the difference of Ago2 expression levels between lung cancer cells and immortalized HBE cells.

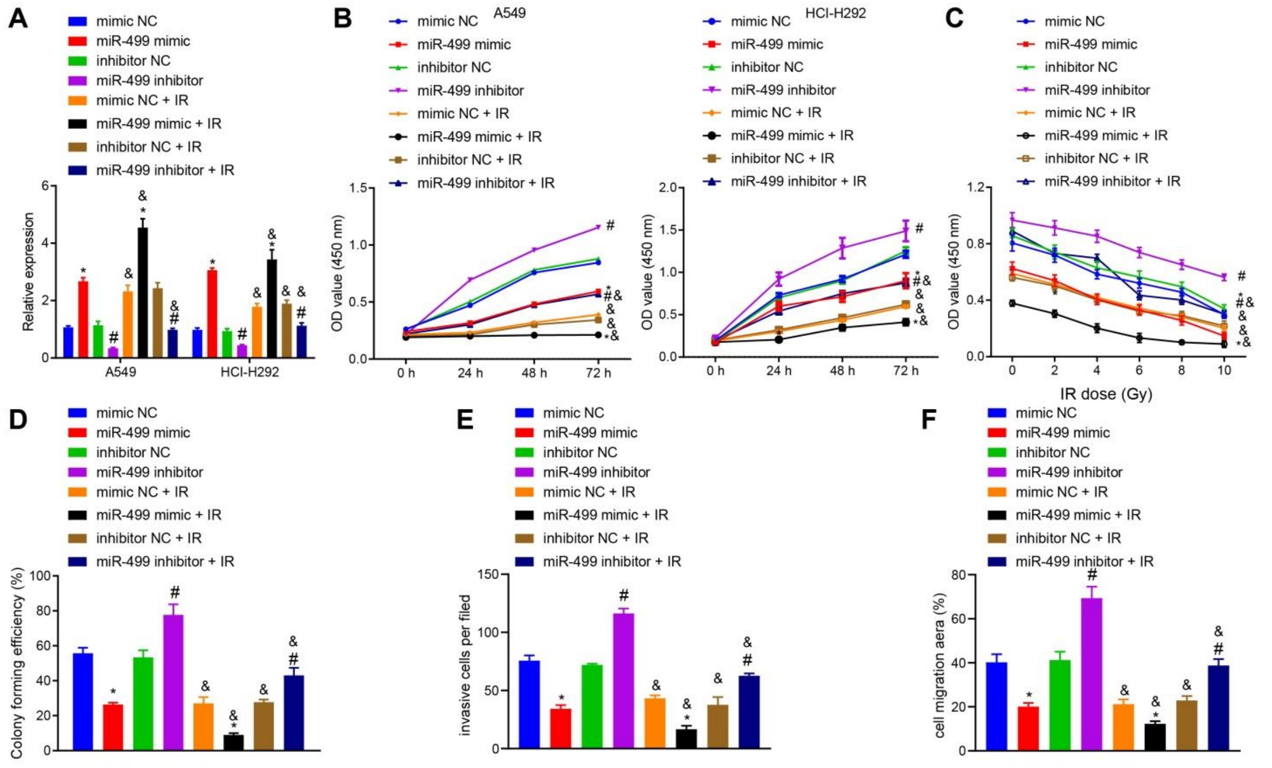

Supplementary Figure 3. A, Western blot was employed to verify the regulatory relationship between CK2α and p65 phosphorylation in A549 cells. B, After 10 Gy irradiation, the effect of miR-499 in A549 cells on radiosensitivity by targeting CK2α was verified by colony formation assay. C, MTT assay was used to detect the sensitivity of lung cancer cells to irradiation. * $p < 0.05$ compared with the miR-499 inhibitor group, and # $p < 0.05$ compared with the inhibitor NC + CX-4945 group. D, Western blot was used to detect the effect of miR-499 on regulating p65, p-p65/p65, Cleaved PARP, caspase-3, and Cleaved caspase-3 expression by targeting CK2α in A549 cells, * $p < 0.05$ compared with the miR-499 inhibitor group and # $p < 0.05$ compared with the inhibitor NC + CX-4945 group. Data were expressed as mean ± standard deviation, independent samples *t*-test was used for comparison between two groups.

Table S2 Primer sequences for RT-qPCR

Gene	Primer sequence (5'-3')	
miR-499	F: TTAAGACTTGCAGTGATGTTT	R: AAACATCACTGCAAGTCTTAA
CK2α	F: TGTTCGTCATGGGTGTGAAC	R: TGGACTGCTTGTGGCTGTGG
GAPDH	F: CCCACTCCTCCACCTTTGAC	R: CATACCAGGAAATGAGCTTGACAA
U6	F: CGACAAGACGATCCGGGTAAA	R: GGTTGAGGAGTGGGTCGAAG

Note: RT-qPCR, reverse transcription quantitative polymerase chain reaction; CK2α, casein kinase 2 alpha; GAPDH, glyceraldehyde-3-phosphate dehydrogenase; F, forward; R, reverse.
