# Supplementary material for: MicroRNA-499 serves as a sensitizer for lung cancer cells to radiotherapy by inhibition of CK2α-mediated phosphorylation of p65
Source: Mol Ther Oncolytics. 2021 Apr 3;21:171–82. doi: 10.1016/j.omto.2021.03.016 (PMC8099482; doi:10.1016/j.omto.2021.03.016)
Supplement: Document S3. Article plus supplemental information [file mmc3.pdf]

Molecular Therapy
Oncolytics
Original Article

MicroRNA-499 serves as a sensitizer for lung cancer cells to radiotherapy by inhibition of CK2α-mediated phosphorylation of p65

Yu-Shui Ma,[1,2,7] Bo-Wen Shi,[3,7] Hai-Min Lu,[4,7] Peng-Fei Xie,[4,7] Rui Xin,[1,7] Zhi-Jun Wu,[5] Yi Shi,[1] Yu-Zhen Yin,[1] Li-Kun Hou,[6] Cheng-You Jia,[1] Wei Wu,[6] Zhong-Wei Lv,[1] Fei Yu,[1] Gao-Ren Wang,[2] Ji-Bin Liu,[2,7] Geng-Xi Jiang,[3] and Da Fu[1]

[1]Department of Nuclear Medicine, Shanghai Tenth People's Hospital, Tongji University School of Medicine, Shanghai 200072, China; [2]Cancer Institute, Nantong Tumor Hospital (Affiliated Tumor Hospital of Nantong University), Nantong 226631, China; [3]Department of Thoracic Surgery, Navy Military Medical University Affiliated Changhai Hospital, Shanghai 200433, China; [4]Department of Thoracic Surgery, Nantong Tumor Hospital (Affiliated Tumor Hospital of Nantong University), Nantong 226631, China; [5]Department of Oncology, Nantong Second People's Hospital, Nantong 226002, China; [6]Department of Pathology, Shanghai Pulmonary Hospital, Tongji University School of Medicine, Shanghai 200433, China

The present study aimed to define the tumor-suppressive role of microRNA-499 (miR-499) in lung cancer cells and its underlying mechanism. First, qRT-PCR analysis revealed poor expression of miR-499 in clinical samples and cell lines of lung cancer. Next, we performed loss- and gain-of-function experiments for the expression of miR-499 in lung cancer cells exposed to irradiation (IR) to determine the effect of miR-499 expression on cell viability and apoptosis as well as tumor growth. Results showed that overexpression of miR-499 inhibited cell viability, enhanced the radiosensitivity of lung cancer cells, and promoted cell apoptosis under IR. Furthermore, CK2α was verified to be a target of miR-499, and miR-499 was identified to repress p65 phosphorylation by downregulating CK2α expression, which ultimately diminished the survival rate of lung cancer cells under IR. Collectively, the key findings of the study illustrate the tumor-inhibiting function of miR-499 and confirmed that miR-499-mediated CK2α inhibition and altered p65 phosphorylation enhances the sensitivity of lung cancer cells to IR.

INTRODUCTION

Lung cancer not only remains one of the most frequently diagnosed cancers but also ranks first for cancer-related mortality, accounting for nearly 2 million deaths worldwide on an annual basis.[1,2] However, approximately 20% to 25% of all lung cancer patients are diagnosed at an early disease stage (stage IA–IIIA), which is amenable to curative surgery.[3] Although various diagnostic procedures are currently available, tissue biopsy remains the mainstay of routine lung cancer diagnostics.[4] This being said, the advancement of prevention strategies and the development of novel treatment strategies are urgently required to improve the rather dismal prognosis of lung cancer.[5] The dysregulation of microRNAs (miRNAs) has been well documented to be associated with the development of lung cancer,[6,7] such as miR-1276,[8] thus highlighting this as a potential treatment target for lung cancer.

In general, miRNAs reportedly play a crucial role in the development of lung cancer by regulating various target genes associated with this malignancy.[9] A previous study suggested that the sequence variation of mature miR-499 conferred an adverse prognosis on lung cancer patients.[10] In addition, serum miR-499 has been suggested to be a promising biomarker for early exploration and prognostic prediction of non-small cell lung cancer (NSCLC).[11] In this regard, we speculated that miR-499 might target CK2α and regulate the development of lung cancer. Interestingly, transcription factors and miRNAs have been reported to have synergistic effects, characterized by unique molecular mechanisms and evolutionary background, which correspond to the two main levels of gene regulatory networks.[12]

Transcription factor CK2α has been reported to contribute to lung cancer metastasis by targeting BRMS1 nuclear output and degradation.[13] Additionally, lung cancer patients have been suggested to benefit from treatment with CK2α inhibitors via activation of the Notch1 signaling pathway.[14] Intriguingly, CK2α has been demonstrated to affect several cellular signaling pathways by phosphorylation mechanisms.[15] Additionally, inhibition of EZH2 by reducing transcription factor p65 presents a new mechanism to suppress human lung cancer cells.[16] Transcription factor p65 also plays a crucial role in the induction of cell anti-viral responses.[17] Based on the aforementioned literature, we

Received 31 October 2020; accepted 31 March 2021;
https://doi.org/10.1016/j.omto.2021.03.016.

[7]These authors contributed equally

Correspondence: Da Fu, PhD, Department of Nuclear Medicine, Shanghai Tenth People's Hospital, Tongji University School of Medicine, Shanghai 200072, China.
E-mail: fu800da900@126.com
Correspondence: Geng-Xi Jiang, PhD, Department of Thoracic Surgery, Navy Military Medical University Affiliated Changhai Hospital, Shanghai 200433, China.
E-mail: jiang9909@hotmail.com
Correspondence: Ji-Bin Liu, PhD, Cancer Institute, Nantong Tumor Hospital (Affiliated Tumor Hospital of Nantong University), Nantong 226631, China.
E-mail: tians2008@163.com

© 2021 The Author(s).
This is an open access article under the CC BY license (http://creativecommons.org/licenses/by/4.0/).

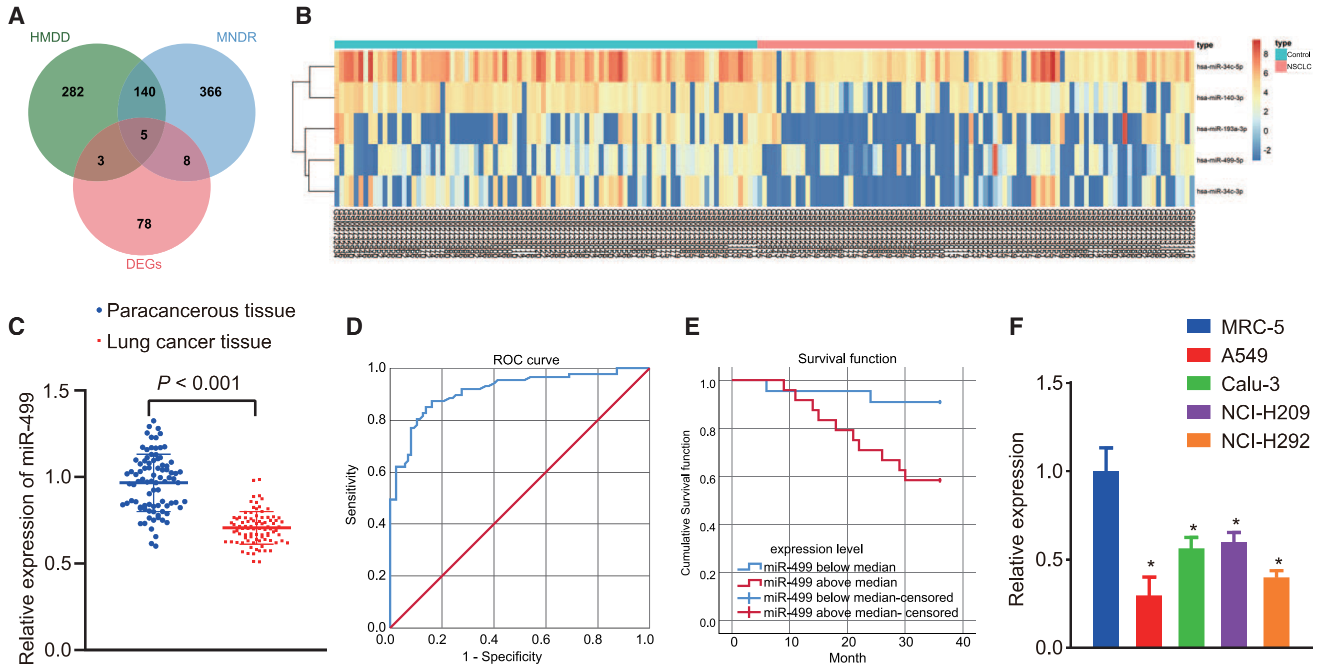

Figure 1. The expression of miR-499 was downregulated in lung cancer and was associated with poor prognosis

(A) Venn diagram displaying the intersection of NSCLC-related miRNAs and differentially expressed miRNAs in HMDD and MNDR databases. (B) Heatmap displaying the expression of candidate miRNAs in control tissues (n = 88) and NSCLC tissues (n = 91). (C) The expression of miR-499 in lung cancer tissues determined by qRT-PCR. (D) The expression of miR-499 in lung cancer cells A549, Calu-3, NCI-H209, and NCI-H292 determined by qRT-PCR. The experiment was repeated at least 3 times independently. (E) ROC analysis for the low and high miR-499 levels in patients with lung cancer, with sensitivity as the ordinate and 1 − specificity as the abscissa. miR-499 produced an area under the curve (AUC) of 0.914 (95% confidence interval [CI] = 0.871 to 0.957), with a sensitivity of 85.1% and a specificity of 86.2% in distinguishing between lung cancer patients and healthy controls. (F) Kaplan-Meier survival analysis based on the miR-499 expression. *p < 0.05 versus adjacent normal tissues or MRC-5 cells or high miR-499 level. The measurement data were expressed as mean ± standard deviation. Comparisons among multiple groups were conducted by ANOVA, followed by Bonferroni's post hoc test. N = 87 for lung cancer tissues and adjacent normal tissues.

designed this study to investigate whether miR-499 could regulate CK2α expression to affect p65 phosphorylation, thereby modulating the sensitivity of lung cancer to irradiation (IR), thus defining new alternative therapeutic strategies for the treatment of lung cancer.

RESULTS

Downregulation of miR-499 is observed in lung cancer and associated with poor prognosis

After differential analysis of the NSCLC-associated miRNA microarray GSE102286, we screened 94 differentially expressed miRNAs (Table S1). Next, we obtained 430 and 519 NSCLC-related miRNAs through the HMDD database (http://www.cuilab.cn/hmdd) and MNDR database (http://www.rna-society.org/mndr/), respectively, and then obtained five candidate miRNAs, namely hsa-miR-34c-5p, hsa-miR-499-5p, hsa-miR-140-3p, hsa-miR-193a-3p, and hsa-miR-34c-3p, after intersection of differentially expressed miRNAs and NSCLC-related miRNAs (Figure 1A). The differential expression heatmap of candidate miRNAs in control and NSCLC samples was also plotted (Figure 1B). It has been reported that miR-499-5p overexpression can inhibit the proliferation and metastasis of NSCLC by targeting VAV3,[18] and that miR-499 overexpression can impart poor prognosis by regulating tumor-related gene expression, enhancing tumorigenesis and chemoresistance. As such, miR-499 may be a relevant biomarker for predicting the prognosis of NSCLC patients.[10]

In order to verify the correlation between miR-499 expression in lung cancer cells and to clarify its relationship with poor prognoses, we analyzed the expression of miR-499 in lung cancer tissues and adjacent normal tissues by quantitative reverse-transcriptase polymerase chain reaction (qRT-PCR). The qRT-PCR results demonstrated that the expression of miR-499 was diminished in the tumor tissues compared with the normal tissues (Figure 1C). For further validation, we selected four human lung cancer cell lines (A549, Calu-3, NCI-H209, and NCI-H292) and one human lung normal cell line (MRC-5) and assessed the expression of miR-499 in these five cell lines by qRT-PCR detection. The results demonstrated that, compared with MRC-5 cells, the expression of miR-499 was reduced in all four cancer cells lines examined (Figure 1D), providing evidence indicating that miR-499 was downregulated in lung cancer cells. Furthermore, receiver operating characteristic (ROC) analysis revealed a cutoff value of 0.798 and indicated that miR-499 could be a diagnostic indicator for patients with lung cancer (Figure 1E). We conducted 2-year follow-up for 12 cases with miR-499 expression above the medium (miR-499-above median) and 75 cases with miR-499 expression below the median value (miR-499-low

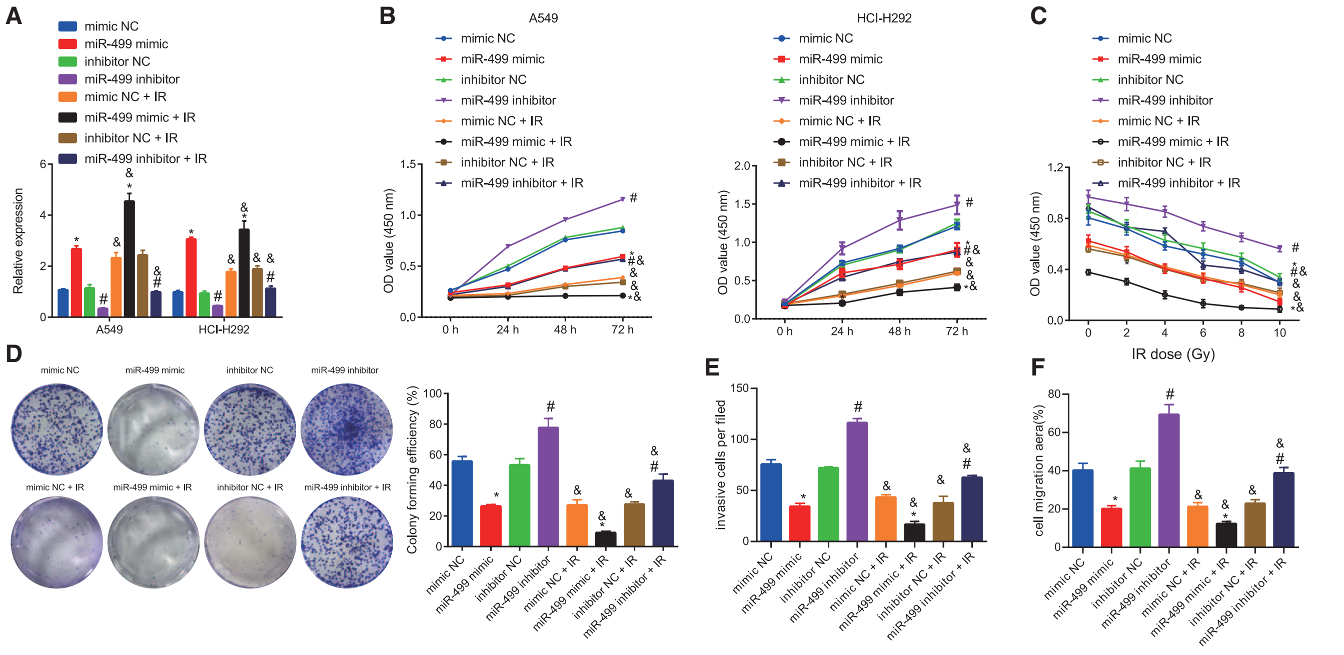

Figure 2. Overexpression of miR-499 elevated the sensitivity of lung cancer cells to IR exposure

(A) The effectiveness of overexpression or inhibition of miR-499 determined by qRT-PCR. (B) The effect of overexpressed or suppressed miR-499 expression on cell viability determined by MTT assay (A549 and NCI-H292 cells). (C) The sensitivity of lung cancer A549 cells with overexpressed or suppressed miR-499 expression to irradiation (IR) determined by MTT assay. (D) The number of colonies of lung cancer A549 cells with overexpressed or suppressed miR-499 expression by colony-formation assay. (E) Cell invasion assay was performed to detect differences in invasion ability of A549 lung cancer cells treated with miR-499 mimic or miR-499 inhibitor. (F) Cell scratch assay was performed to detect differences in migration ability of A549 lung cancer cells treated with miR-499 mimic or miR-499 inhibitor. *p < 0.05 versus cells treated with mimic NC; #p < 0.05 versus cells treated with inhibitor NC, n = 3; &p < 0.05 versus the corresponding non-irradiated groups, n = 3. The measurement data were expressed as mean ± standard deviation. Data between the two groups were compared using independent sample t test. Comparisons among multiple groups were conducted by ANOVA, followed by Bonferroni's post hoc test. Statistical analysis in relation to different concentrations within each group was analyzed using two-way ANOVA, followed by Bonferroni's post hoc test. The experiments were repeated three times, with the representative result presented.

median). Finally, Kaplan-Meier survival analysis of the overall survival of patients was performed as a function of the tumor level of miR-499 expression. The results illustrated that lung cancer patients with high expression of miR-499 exhibited a higher overall survival rate (Figure 1F).

Overexpression of miR-499 enhances the sensitivity of lung cancer cells to IR exposure *in vitro*

We further found that the expression of miR-499 was elevated in miR-499 mimic-IR-treated cells relative to miR-499 mimic-treated cells, in inhibitor negative control (NC)-IR-treated cells compared to inhibitor NC-treated cells, and in miR-499 inhibitor-IR-treated cells compared to miR-499 inhibitor-treated cells (Figure 2A). We subsequently overexpressed or knocked down miR-499 using miR-499 mimic or miR-499 inhibitor in NCI-H292 cells. The 3-(4,5-dimethylthiazol-2-yl)-2,5-diphenyltetrazolium bromide (MTT) assay revealed that the cell viability in the miR-499 mimic group was significantly reduced compared with the mimic NC group and that cell viability in the miR-499 inhibitor group was remarkably increased compared with the inhibitor NC group. Furthermore, compared with the corresponding non-irradiated groups (mimic NC-IR versus mimic NC, miR-499 mimic-IR versus miR-499 mimic, inhibitor NC-IR versus inhibitor NC, miR-499 inhibitor-IR versus miR-499 inhibitor), the proliferation rate of cells with different treatments was significantly reduced following IR (Figure 2B). Meanwhile, we evaluated the viability of A549 (Figure 2C) and NCI-H292 (Figure S1A) cells via the MTT assay after 1–10 Gy IR exposure. The cell viability after miR-499 mimic treatment was diminished with identical doses of IR, while cell viability was elevated after miR-499 inhibitor treatment under the same doses of IR. In addition, compared with the corresponding non-irradiated groups (mimic NC-IR versus mimic NC, miR-499 mimic-IR versus miR-499 mimic, inhibitor NC-IR versus inhibitor NC, miR-499 inhibitor-IR versus miR-499 inhibitor), the proliferation rate following IR of cells with different treatments was significantly reduced. Finally, colony formation abilities in A549 (Figure 2D) and NCI-H292 (Figure S1B) cells displayed a reduced cell colony formation upon treatment of miR-499 mimic and elevated cell colony formation following treatment with miR-499 inhibitor after 10 Gy IR exposure; compared with the corresponding non-irradiated groups (mimic NC-IR versus mimic NC, miR-499mimic-IR versus miR-499 mimic, inhibitor NC-IR versus inhibitor NC, miR-499 inhibitor-IR versus miR-499 inhibitor), the colonies of cells with different treatments were significantly reduced following IR. Moreover, we found that the invasion and migration abilities of miR-499 mimic-treated cells were decreased

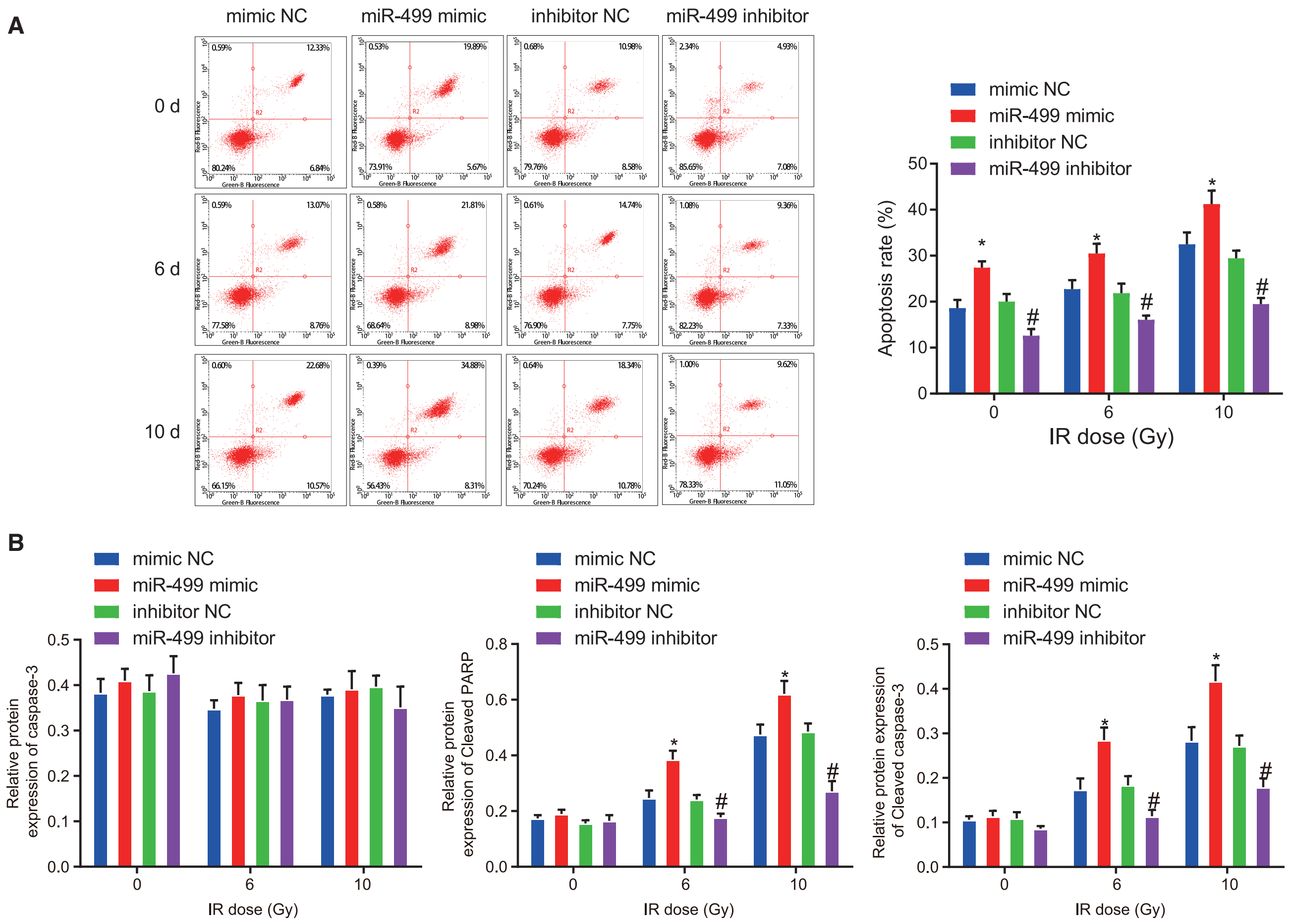

Figure 3. miR-499-enhanced IR-induced apoptosis of lung cancer A549 cells *in vitro*

(A) The effect of overexpression or silencing of miR-499 on IR-induced apoptosis detected by flow cytometry assay. (B) The effect of overexpression or silencing of miR-499 on IR-induced caspase-3, cleaved PARP, and cleaved caspase-3 protein expression determined by western blot analysis. *p < 0.05 versus cells treated with mimic NC; #p < 0.05 versus cells treated with inhibitor NC. The measurement data were expressed as mean ± standard deviation. Data between the two groups were compared using independent sample t test. The experiments were repeated for three times, with the representative result presented.

compared with the mimic NC-treated cells, but the invasion and migration abilities of miR-499 inhibitor-treated cells were significantly increased compared with the inhibitor NC-treated cells. Compared with the corresponding non-irradiated experimental group (mimic NC-IR versus mimic NC, miR-499 mimic-IR versus miR-499 mimic, inhibitor NC-IR versus inhibitor NC, miR-499 inhibitor-IR versus miR-499 inhibitor), the invasion and migration abilities of cells with different treatment was decreased following IR (Figures 2E and 2F). The aforementioned results demonstrated that miR-499 expression was negatively correlated with the growth of cancer cells and positively correlated with the sensitivity of lung cancer cells to IR exposure.

miR-499 promotes IR-induced apoptosis of lung cancer cells *in vitro*

Flow cytometry assay was employed to detect apoptosis in A549 (Figure 3A) and NCI-H292 (Figure S1C) cell lines after varying doses of IR (0, 6, 10 Gy) in response to miR-499 mimic/inhibitor. Without IR exposure, apoptosis was augmented in miR-499 mimic-transfected cells, while it was reduced upon miR-499 inhibitor transfection. Apoptosis was enhanced in all the groups following IR exposure, but a higher rate of apoptosis was observed in the cells transfected with miR-499 mimic when compared to cells transfected with mimic NC. Relative to the inhibitor NC-treated A549 cells, the apoptosis was decreased in miR-499 inhibitor-treated A549 cells. Higher doses of IR led to a more pronounced difference in apoptosis between miR-499 mimic- and mimic NC-treated cells. Meanwhile, western blot analysis of the protein expression apoptosis-related factors in A549 (Figure 3B) and NCI-H292 (Figure S1D) cell lines further validated the aforementioned results. A549 cells treated with miR-499 mimic displayed no change in caspase-3 expression but elevated expression of cleaved PARP and cleaved caspase-3. In addition, higher IR doses resulted in greater increases in the expression of cleaved PARP and cleaved caspase-3. On the other hand, miR-499 inhibitor transfection exerted no effect on caspase-3 expression but resulted in reduced expression

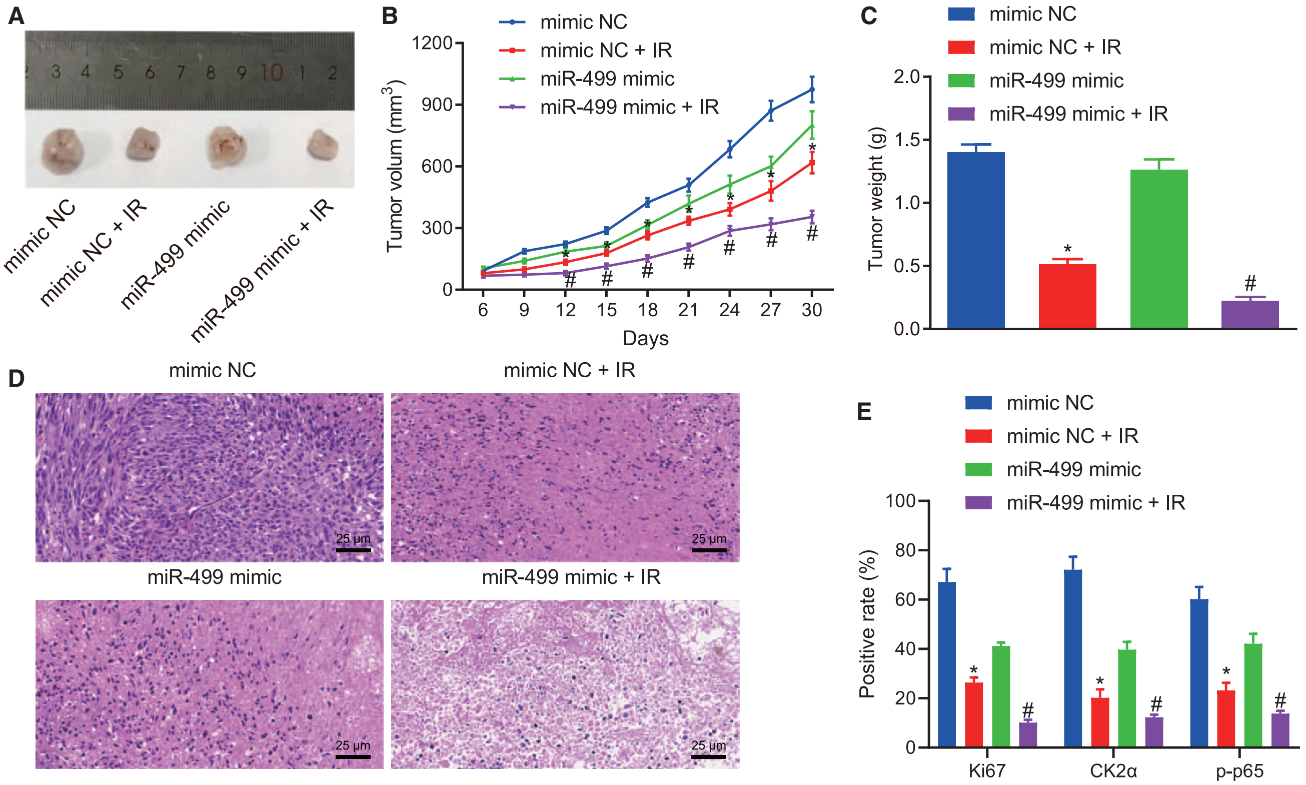

Figure 4. miR-499 increased the sensitivity of lung cancer A549 cells to IR exposure *in vivo*

(A) Representative pictures of tumors after different treatment. (B) Measurements of tumor volume after overexpression of miR-499 and IR. (C) Measurements of tumor weight after overexpression of miR-499 and IR. (D) H&E staining was conducted to detect the cellular structure changes (×400). (E) Ki67, CK2α, and p-p65 expression in tissues detected by IHC. *$p < 0.05$ versus mice treated with mimic NC; $^{\#}p < 0.05$ versus mice treated with mimic NC + IR, n = 12. The measurement data were expressed as mean ± standard deviation. Data between two groups were compared using independent sample t test. Comparisons among multiple groups were conducted by ANOVA, followed by Bonferroni's post hoc test.

of cleaved PARP and cleaved caspase-3. Furthermore, higher IR doses attenuated the decreases in expression of cleaved PARP and cleaved caspase-3. Therefore, we conclude that overexpression of miR-499-enhanced IR-induced apoptosis of lung cancer cells.

miR-499 increases the sensitivity of lung cancer cells to IR exposure *in vivo*

We subsequently subcutaneously injected A549 cell lines transfected with miR-499 mimic into the nude mice. The nude mice were subjected to 10 Gy IR on the ninth day after xenografting and photographed every 3 days to monitor the tumor growth of different groups (Figures 4A–4C). The *in vivo* experiments demonstrated that, in comparison to the mice injected with mimic NC, there was a slight decrease in tumor volume and weight in mice injected with miR-499 mimic, and, furthermore, the tumor volume and weight of mice treated with mimic NC + IR were reduced. In comparison with mice treated with mimic NC + IR, the tumor volume and weight were notably diminished in mice treated with miR-499 mimic and IR. Thus, miR-499 overexpression augmented the sensitivity of lung cancer cells to IR exposure *in vivo*. The results of hematoxylin and eosin (H&E) staining assay of the resected tumors showed that, compared with the mimic NC group, the cytoplasmic content of some cells in the miR-499 mimic group was concentrated, along with absent nucleoli, and likewise in the mimic NC + IR group. Compared with the mimic NC + IR group, cells in the miR-499 mimic + IR group had the most significant shrinkage, absence of nucleoli, and cell necrosis (Figure 4D). The results of immunohistochemistry (IHC) showed that the number of Ki67-, CK2α-, and p-p65-positive cells was significantly decreased in the miR-499 mimic group compared with the mimic NC group, and the number of Ki67-, CK2α-, and p-p65-positive cells was significantly reduced in the mimic NC + IR group. Compared with the mimic NC + IR group, the number of Ki67-, CK2α-, and p-p65-positive cells in the miR-499 mimic + IR group was significantly reduced (Figure 4E).

miR-499 upregulates the sensitivity of lung cancer cells to IR exposure via inhibition of CK2α to repress p65 phosphorylation

We had first predicted 600, 1,461, and 1,576 downstream target genes of miR-499 by scrutiny of the bioinformatics databases TargetScan, mirDIP, and RNAInter, respectively, and also obtained 2,856 NSCLC-related genes by GeneCards database. Next, 58 candidate genes were flagged by intersection of target genes with NSCLC-related genes (Figure 5A). The candidate gene interaction relationship

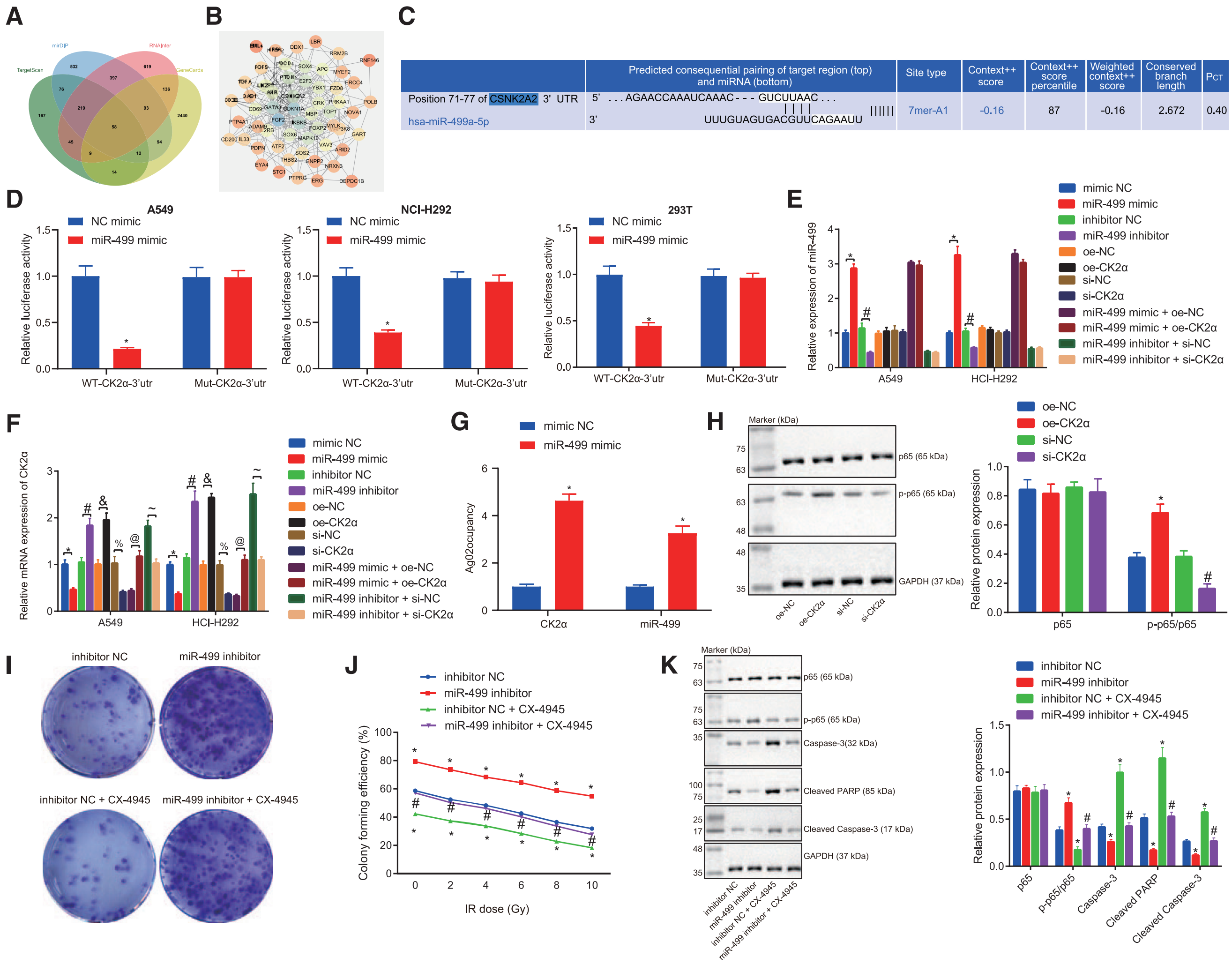

Figure 5. miR-499 promoted the sensitivity of lung cancer cells to IR exposure by targeting CK2α and blocking p65 phosphorylation

(A) Venn diagram displaying the intersection of downstream target gene results of miR-499 predicted by TargetScan, mirDIP, and RNAInter bioinformatics databases and NSCLC-related genes in GeneCards database. (B) STRING was used to analyze the network diagram of interaction relationships between candidate genes, with circles ranging in color from orange to blue indicating the increasing degree of genes and lines in the middle of circles indicating interaction relationships between genes. (C) Prediction of relationship between miR-499 and CK2α by a bioinformatics website. (D) Dual-luciferase activity in HEK293T cells detected by dual-luciferase gene assay. (E and F) The regulatory relationship between miR-499 and CK2α verified by qRT-PCR. *p < 0.05 versus cells treated with NC mimic; #p < 0.05 versus cells transfected with inhibitor NC (E and F); &p < 0.05 versus upon treatment of oe-NC; %p < 0.05 versus upon treatment of si-NC; @p < 0.05 versus cells treated with miR-499 mimic and oe-NC; ~p < 0.05 versus cells transfected with miR-499 inhibitor and si-NC. (G) RIP assay verifying the binding of miR-499 to CK2α. (H) The regulatory relationship between CK2α and p65 phosphorylation verified by western blot analysis in A549 cells. (I) Sensitivity of lung cancer cells to radiation as determined by MTT assay. *p < 0.05 versus cells treated with oe-NC; #p < 0.05 versus cells transfected with si-NC. (J) The effect of miR-499 through targeting CK2α on the sensitivity of radiotherapy after 10 Gy IR verified by colony-formation assay in A549 cells. *p < 0.05 versus cells treated with miR-499 inhibitor; #p < 0.05 versus cells treated with inhibitor NC + CX-4945. (K) The effects of miR-499 on p65, phosphorylated p65/p65, cleaved PARP, caspase-3, and cleaved caspase-3 expression determined by western blot analysis in A549 cells. *p < 0.05 versus cells treated with miR-499 inhibitor; #p < 0.05 versus cells treated with inhibitor NC + CX-4945. The measurement data were expressed as mean ± standard deviation. Data between two groups were compared using independent sample t test.

network was obtained by the STRING tool, and the interaction relationship network of the genes was plotted. The results showed that five genes, namely FGF2, CDKN1A, GATA3, IKBKB, and CSNK2A2 (alias: CK2α), were in the core position (degree ≥ 17) (Figure 5B). Further, we found that CK2α knockout can significantly enhance the radiosensitivity of a variety of lung cancer cells.[19] Binding sites between miR-499 and CK2α were predicted and subsequently verified via the bioinformatics website (http://www.targetscan.org/mamm_31/) and confirmed by dual luciferase gene assay in HEK293T and A549 cells, which showed that CK2α could indeed augment the tolerance of lung cancer cells to radiotherapy (Figures 5C and 5D). qRT-PCR results (Figures 5E and 5F) demonstrated

that, in comparison to the cells treated with mimic NC, miR-499 expression was increased, while the expression of CK2α was diminished in the cells treated with miR-499 mimic. In comparison to the cells transfected with inhibitor NC, miR-499 expression was reduced, while the expression of CK2α was markedly elevated in the cells transfected with miR-499 inhibitor. In comparison to oe-NC treated cells, there was no significant difference detected in the expression of miR-499, whereas the expression of CK2α was elevated in oe-CK2α-treated cells. Relative to the si-NC-transfected cells, CK2α expression was notably diminished in the si-CK2α-transfected cells. In the miR-499 mimic and oe-CK2α-treated cells, there was a significant increase in CK2α expression relative to treatment with miR-499 mimic and oe-NC. In the cells treated with miR-499 inhibitor and si-CK2α, there was a considerable reduction in CK2α expression observed in comparison to cells treated with miR-499 inhibitor and si-NC.

miRNAs exist in the cytoplasm as components of the RNA-induced silencing complex (RISC), of which Ago2 is a key component that is required for miRNA-mediated gene silencing.[20] The results of qPCR and western blot analyses showed no significant difference in the expression levels of Ago2 between cancer tissues and adjacent normal tissues, as well as between lung cancer cells and immortalized human bronchial epithelial cell line cells (Figure S2). In this study, an RNA binding protein immunoprecipitation (RIP) experiment was performed in lung-cancer-resistant cell extracts using an antibody against Ago2 to determine whether CK2α and miR-499 belong to the same RISC. RIP results showed that miR-499 mimic-treated cells had Ago2-rich miRNAs compared with control immunoglobulin G (IgG) immunoprecipitation. High CK2α expression was detected in the same precipitate (Figure 5G).

p65 expression and phosphorylation of p65 content was analyzed in A549 cells by western blot after overexpression or silencing of CK2α. The results revealed no notable change in the expression of p65, while the relative phosphorylation of p65 content was elevated in cells transfected with oe-CK2α compared with cells transfected with oe-NC (Figure 5H). In comparison to the treatment with si-NC, there was no significant change of p65 expression, while the relative phosphorylation of p65 content was remarkably reduced in the cells with si-CK2α treatment (Figure 5H).

Next, to verify further whether miR-499 could increase the sensitivity to radiotherapy by targeting CK2α to regulate p65 phosphorylation, we treated lung cancer A549 cells with a CK2α inhibitor (CX-4945) following the inhibition of miR-499. Cell survival rate was determined using colony-formation assay after IR exposure at different doses (0, 2, 4, 6, 8, and 10 Gy), and the expressions of the related proteins were analyzed by western blot analysis. After 10 Gy IR exposure, the higher doses of IR resulted in lower cell survival rate. Compared with inhibitor NC treatment, the cell survival rate increased in the presence of miR-499 inhibitor but decreased in cells treated with CX-4945. In addition, cell survival rate was increased in response to miR-499 inhibitor and CX-4945 treatment compared to CX-4945 alone (Figure 5I). Cell survival in different groups was detected by colony-formation assay. The results showed that the colony formation was significantly reduced with increasing IR dose; compared with the inhibitor NC group, the cell colonies in the miR-499 inhibitor group were significantly increased, whereas the cell colonies in the inhibitor NC + CX-4945 group were significantly reduced. Compared with the inhibitor NC + CX-4945 group, the miR-499 inhibitor + CX-4945 group had significantly increased colonies (Figure 5J).

Following miR-499 inhibitor treatment, there was no significant difference in p65 expression; the relative phosphorylation of p65 content was elevated, while the expression of cleaved PARP, caspase-3, and cleaved caspase-3 was diminished in comparison to inhibitor NC treatment. Furthermore, while inhibitor NC + CX-4945 treatment exerted no effect on p65 expression, it decreased phosphorylation of p65 content and elevated the expression of cleaved PARP, caspase-3, and cleaved caspase-3. Relative to miR-499 inhibitor treatment, miR-499 inhibitor and CX-4945 treatment exerted no effect on p65 expression but resulted in reduced phosphorylation of p65, as well as elevated expression of cleaved PARP, caspase-3, and cleaved caspase-3. Together, these results provided evidence demonstrating that miR-499 could target CK2α to inhibit p65 phosphorylation and repress apoptosis (Figure 5K). The same results were also obtained in NCI-H292 cells (Figures S3A–S3D). Altogether, the aforementioned results indicated that targeted regulation of miR-499 could potentially be used as a treatment to prevent the spread of lung cancer and the onset of radioresistance.

DISCUSSION

A diverse group of miRNAs continues to be implicated in the occurrence and development of lung cancer.[21] Thus, the overexpression of miR-499 has been previously reported to repress the proliferation and metastasis of NSCLC.[22] The present study further evidenced that miR-499 overexpression could inhibit the development of lung cancer and enhance the sensitivity of lung cancer cells to IR exposure.

The results obtained demonstrated low levels of miR-499 expression in lung cancer tissues and cells compared to non-cancer samples. In addition, the forced overexpression of miR-499 resulted in elevated radiotherapy sensitivity of lung cancer cells *in vitro*. Overexpression of miR-499 was found to repress proliferation while elevating the apoptosis of lung cancer cells. Existing literature has suggested that the knockdown of miR-499 could augment apoptosis at the later stages of cell differentiation.[23] Previous research has indicated that miR-499a may enhance the apoptosis of glioma cells by inhibiting the MAPK signaling pathway.[24] The aforementioned findings were partially consistent with our earlier observations that miR-499 contributed to the apoptosis of lung cancer cells. Furthermore, the overexpression of miR-499 enhanced the sensitivity of lung cancer cells to IR, decreased cell viability, and promoted cell apoptosis under different doses of IR, as well as increasing the levels of the apoptosis-related proteins cleaved PARP and cleaved caspase-3. Additionally, apoptosis was reflected by the regulation of markers such as the elevated level of cleaved-caspase 3 and cleaved-PARP proteins.[25] Interestingly, miR-499-5p has been reported to trigger an increase in the expression levels of Bcl-2 and downregulate the expression of caspase-3.[26]

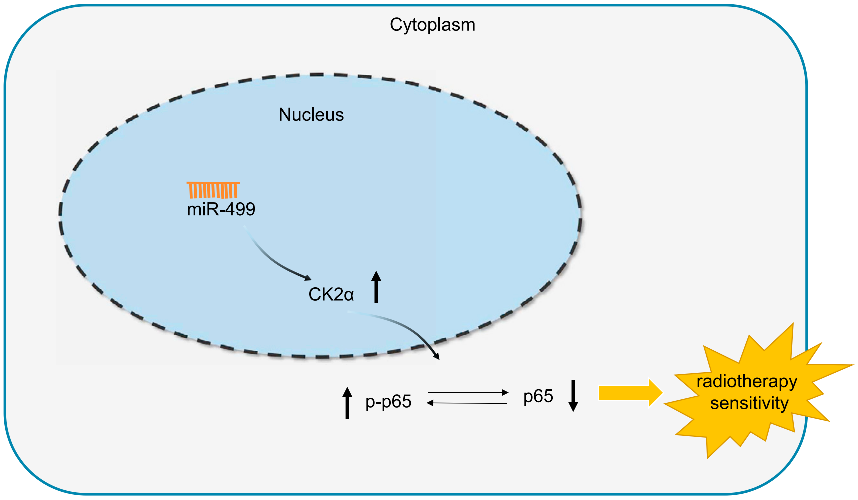

Figure 6. The molecular mechanism of the regulatory network and function of miR-499

Overexpression of miR-499 increases the sensitivity of lung cancer cells to IR exposure by targeting CK2α and suppressing p65 phosphorylation.

In the subsequent experiments, bioinformatics website analysis and dual-luciferase gene assay were performed to predict and validate the relationship between miR-499 and CK2α. We then uncovered that miR-499 targeted and inhibited CK2α, thus contributing to elevated sensitivity of lung cancer cells to IR exposure. The upregulation of miR-760 and miR-186 has been suggested to induce replicative senescence in human lung fibroblast cells by CK2α.[27] Additionally, a previous study also demonstrated that the inhibition of miR-125b could target CK2α to ameliorate cerebral ischemia/reperfusion injury.[28] Transcription factors are DNA-binding proteins that bind to transcription co-regulators, ultimately leading to histone modifications capable of altering chromatin structure to regulate gene transcription.[29] CK2α has been speculated to be a promising target for enhancing the radio-sensitivity in NSCLC.[19] Our investigation demonstrated that CK2α could augment lung cancer inhibition or could aggravate the sensitivity of lung cancer cells to IR exposure. Numerous previous studies have evidenced that miR-499 could inhibit p65 phosphorylation by targeting CK2α to heighten radiosensitivity of cell lines. Phosphorylation is the prototypical post-translational modification of key proteins, which is capable of influencing diverse physiological functions of cells.[30] Accordingly, we have considered the possibility that the survival of CK2 in prostate cancer may be mediated by the maintenance and promotion of androgen receptor and NF-κB p65 expression.[31] Moreover, the ectopic expression of miR-506 has also been demonstrated to repress the expression of NF-κB p65 and to subsequently activate p53, resulting in the inhibition of lung cancer cell survival,[32] which seems partially consistent with the present findings. Furthermore, diminished levels of p65 expression may enhance the role of solamargine in human lung cancer cells.[33]

Taken together, the key findings of the present study highlight that miR-499 may target and downregulate CK2α expression to repress p65 phosphorylation, ultimately contributing to the enhancement of lung cancer cell sensitivity to IR exposure (Figure 6). Our findings emphasize the potential of miR-499 as a promising therapeutic target for enhancing radiotherapy in lung cancer treatment. However, this study has certain limitations, which call for further clinical and biological verifications of the therapeutic action of miR-499 in lung cancer.

MATERIALS AND METHODS

Ethics statement

The study protocols were performed with the approval of the Ethics Committee of Shanghai Tenth People's Hospital, Tongji University School of Medicine and conducted in strict accordance with the Helsinki Declaration. All the participants signed informed written consent documentation. All animal experiments were performed in compliance with the recommendations in the Guide for the Care and Use of Laboratory Animals of the National Institutes of Health.

Bioinformatics analysis

Through the GEO database (https://www.ncbi.nlm.nih.gov/geo/), the NSCLC-associated miRNA microarray GEO: GSE102286 was obtained, which included 88 normal samples and 91 NSCLC samples. The R language "limma" package (http://www.bioconductor.org/packages/release/bioc/html/limma.html) was used for differential analysis of microarray miRNA expression profiles with $|\log2FC| > 1$ and $p < 0.05$ as screening criteria of differentially expressed miRNAs. Using the R language "pheatmap" package (https://cran.r-project.org/web/packages/pheatmap/index.html), the differential expression heatmap of candidate miRNAs was plotted. Through the HMDD database (http://www.cuilab.cn/hmdd), the MNDR database (http://www.rna-society.org/mndr/), and a literature search, NSCLC-associated miRNAs were obtained. By using the jvenn tool (http://jvenn.toulouse.inra.fr/app/example.html), the overlapping parts of differentially expressed miRNAs and NSCLC-related miRNAs were obtained. Through the bioinformatics database TargetScan (http://www.targetscan.org/vert_72/), mirDIP (http://ophid.utoronto.ca/mirDIP/, minimum score = high), and RNAInter (http://www.rna-society.org/rnainter/, score > 0.17), the downstream target genes of miRNAs were predicted. NSCLC-related genes were retrieved through the GeneCards database (https://www.genecards.org/, score > 7), and the jvenn online tool (http://jvenn.toulouse.inra.fr/app/example.html) was employed to search for the overlapping parts of predicted miRNA target genes with NSCLC-related genes. By the STRING tool (https://string-db.org/), the interaction between candidate targets was analyzed, the interaction relationship network of genes was visualized using Cytoscape 3.5.1 software, and the node and edge of the network were analyzed using the network analyzer built-in tool to obtain the

degree of the gene (degree). To further predict the downstream regulators of target genes, we used the BioGRID database (https://thebiogrid.org/, evidence $\geq$ 2) to search for the interaction factors of genes and intersect them with NSCLC-related genes in the GeneCards database to screen potential targets. Finally, the Phenolyzer tool (http://phenolyzer.wglab.org/) was used to obtain the correlation ranking and score of candidate genes with NSCLC.

Study subjects

Lung cancer tissues (n = 87) and adjacent normal tissues (n = 87) were collected from 87 lung cancer patients who had undergone lung cancer resection at the Shanghai Tenth People's Hospital, Tongji University School of Medicine from June 2013 to April 2016. Lung cancer tissues and adjacent normal tissues were fixed with formalin and embedded in paraffin for histopathological diagnosis. Adjacent normal tissues were confirmed by tissue biopsy ($\geq$5 cm away from cancer tissues). All the patients were followed up for a period of at least 3 years, with follow-up ending in April 2019.

qRT-PCR

Total RNA was extracted from the tissues or cells using a TRIzol kit (15596-018; Beijing Solarbio Science & Technology, Beijing, China) in accordance with the manufacturer's instructions, with RNA concentration determined accordingly. The primers applied in this study were synthesized by Dalian Takara (Dalian, China) (Table S2). Reverse transcription was subsequently performed using a complementary DNA (cDNA) Reverse Transcription Kit (K1622, Beijing Yaanda Biotechnology, Beijing, China) in accordance with the manufacturer's instructions. The reversely transcribed cDNA was diluted to 50 ng/μL for fluorescent qPCR in a real-time PCR instrument (ViiA 7, Daan Gene of Sun Yat-sen University, Guangzhou, China). U6 and glyceraldehyde-3-phosphate dehydrogenase (GAPDH) were regarded as internal references. The relative transcription level of the target gene was calculated by relative quantification ($2^{-\triangle\triangle CT}$ method).

Western blot analysis

Total protein was extracted from tissues or cells via the addition of phenylmethylsulphonyl fluoride (PMSF) and protease inhibitors. The protein concentration of each sample was determined using a bicinchoninic acid (BCA) kit (23227, Thermo Fisher Scientific, Waltham, MA, USA). The protein was separated by polyacrylamide gel electrophoresis and then transferred onto a polyvinylidene fluoride (PVDF) membrane, which was then blocked with 5% bovine serum albumin (BSA) for 1 h at ambient temperature. The membrane was then incubated at 4°C overnight with rabbit primary antibodies against CK2α (1:1,000, 702811, Thermo Fisher Scientific), p65 (1:1,000, ab16502, Abcam, Cambridge, UK), phosphorylated p65 (1:1,000, A7169, Assay Biotech Trading Partners, Encinitas, CA, USA), caspase 3 (1:5,000, ab32351, Abcam), cleaved PARP (1:5,000, ab32561, Abcam), cleaved caspase-3 (1:5,000, ab32042, Abcam), and β-actin (1:5,000, ab75186, Abcam). The membrane was washed three times (5 min per wash) using Tris buffered saline Tween (TBST), followed by incubation with horseradish peroxidase (HRP)-labeled goat anti-rabbit IgG (1:20,000, ab205718, Abcam) for 1.5 h at ambient temperature. After incubation, the membrane was washed three times (5 min per wash) in TBST and developed using a developer (NCI4106, Pierce, Rockford, IL, USA). ImageJ 1.48u software (Bio-Rad, Hercules, CA, USA) was employed for protein quantitative analysis (gray ratio of each protein and internal reference β-actin).

MTT assay

A single-cell suspension was prepared using culture medium containing 10% fetal bovine serum (FBS) and seeded into a 96-well plate at a volume of 200 μL/well and cultured for 3–5 days. Next, 20 μL of MTT solution was added to the cells for incubation for an additional 4 h. The culture was terminated with the supernatant in the well discarded. Next, 150 μL of dimethyl sulfoxide (DMSO) was added to each well followed by shaking for 10 min. The absorbance of each well was subsequently determined at 490 nm on the enzyme-linked immunosorbent monitor, and the results were recorded. The cell growth curve was then plotted with time as the abscissa and absorbance as the ordinate.

Colony-formation assay

When the cells reached approximately 70% confluence, a cell-colony-formation assay test was performed. RPMI 1640 medium containing 10% FBS was mixed with 10% agarose gum solution in a ratio of 1:10. More specifically, 5 mL of the mixture was placed in a sterile Petri dish and allowed to solidify and subsequently preserved in a 37°C, 5% CO_2 incubator for later use. The cells were initially digested with 0.25% trypsin into single cells, centrifuged, dispersed with 10% FBS and RPMI 1640, counted and diluted to a cell density of 500 cells/mL. Next, 2% agarose gum solution was mixed with cell suspension at a ratio of 10:1, followed by the addition of 2 mL of the mixture to the prepared agarose, and placed in a 37°C, 5% CO_2 incubator for 7 days. The formation of the cell colonies was then observed and the colony-formation assay rate (clone formation rate = number of formed clones/number of cells inoculated) was calculated.

Flow cytometry assay

Following a 24 h period of transfection, apoptosis of the lung cancer cells was detected by Annexin V fluorescein isothiocyanate (FITC)/propidium iodide (PI) double-staining kit (556547, Shanghai Solja Technology, Shanghai, China). In brief, 10× binding buffer was diluted into 1× binding buffer with deionized water. Cells were digested and dispersed into single cells, followed by centrifugation at 2,000 rpm for 5 min at ambient temperature for cell collection. The cells were then resuspended with pre-cooled 1× PBS, centrifuged at 2,000 rpm for 5–10 min, and suspended by 300 μL of 1× binding buffer. The cells were then mixed with 5 μL of Annexin V-FITC and incubated for 15 min at ambient temperature in the dark. The cells were added with 5 μL of PI and protected from light for 5 min in an ice bath 5 min prior to analysis with a flow cytometer (Cube 6, Partec, Munster, Germany). The excitation wavelength was set at 419 nm, with FITC detected at 519 nm and PI detected at a wavelength greater than 575 nm.

Wound-healing assay

Cells from each group in the logarithmic phase were collected and seeded in 6-well plates for culture at 10^6 cells/well. A marker pen was used to evenly draw lines on the back of the 6-well plates, which were used as the positioning for photographic recording. After the cells were completely distributed on the surface of the 6-well plates, the original culture medium was replaced with cell culture medium containing 1% FBS, and the cells were starved for 12 h. With the help of a ruler, a 200 μL pipette tip was used to scratch vertical lines on the 6-well plate. The cells were washed three times with 2 mL of PBS, and the scratched cells were washed off and treated as described above followed by photographing at 0 and 24 h. Cell migration rate (%) was calculated as (1 − scratch width/initial scratch width) × 100%. SPSS software was used for statistical analysis of data at each time point in each group.

Transwell assay

Tubes and pipette tips were pre-cooled at −20°C before testing. Basement membranes of the Transwell chambers were coated with Matrigel glue (356234, Corning, Corning, NY, USA) diluted with serum-free cold DMEM cell medium. A total of 100 μL of the diluted gel was added to the upper chamber of a 24-well Transwell, which was incubated at 37°C for at least 4–5 h. The basement membrane was hydrated, and the gel was gently washed with serum-free medium. The cells were then detached and washed three times with culture medium. Cells were resuspended in a density of 5 × 10^5 cells/mL with 1% FBS. Next, a total of 200 μL of cell suspension was added to the upper chamber, and 600 μL of cell culture medium was added to the lower chamber. After incubation at 37°C for 20–24 h, a cotton swab was used to wipe off the non-invasive cells on the upper chamber. Transwell chambers were removed from the incubator and washed twice with PBS for 2 min each. The cells were then fixed with methanol and acetic acid at a ratio of 3:1 for 15–30 min and dried. Next, 500 μL solution containing 1% crystal violet was added to a 24-well plate, and the chamber membranes were immersed in the culture medium. The membrane was removed after 30 min at 37°C and washed with PBS. Four visual fields were photographed, and the number and diameter of migrated cells were counted.

Tumor formation in nude mice

Forty-eight BALB/c nu/nu mice (aged 4–6 weeks; weighing 12–19 g; 24 male mice and 24 female mice; Shanghai Lingchang Systems, Shanghai, China) were raised in the specific-pathogen-free (SPF) environment in the Animal Experimental Center (Experimental Animal Qualification certificate no. 159) of Shanghai Tenth People's Hospital, Tongji University School of Medicine prior to the commencement of experimental procedures. The mice were acclimatized for 7 days and provided free access to food and drinking water in a 12 h light-dark cycle in aseptic conditions. The mice were then subcutaneously injected through the dorsal area with commercially purchased lung cancer A549 and NCI-H292 cell lines, which were transfected with mimic NC, miR-499 mimic, and/or subjected to IR exposure, with 12 mice in each group. After inoculation, the mice were placed in a laminar flow hood in the SPF animal room. Tumor growth was observed every 3 days following inoculation and recorded for 30 days. The tumors were then removed and weighed.

H&E staining

After animal dissection, the tumors were placed in PBS at pH 7.4 for 2 h. Next, the tumors were dehydrated in a graded alcohol system, embedded in paraffin, and cut to a thickness of 5 μm before staining with H&E.

IHC

Sections were prepared from the rehydrated paraffin-embedded tissues using IHC, and the sections were incubated with antibodies against Ki67 (1:200, ab15580, Abcam, UK), p-p65 antibody (1:200, ab31624, Abcam, UK), and CK2α (1:200, PA5-28686, Thermo Scientific, Waltham, MA, USA). Sections were then stained with biotinylated anti-mouse and anti-rabbit secondary antibodies to IgG (1:200; Sigma-Aldrich) for 2 h and incubated with HRP-conjugated streptavidin for 1 h. Images were taken using a Leica DMI4000B microscope (Olympus Soft Imaging Solutions, Hamburg, Germany).

RIP

The binding of miR-499 with CK2α was detected using the RIP kit (Millipore, Burlington, MA, USA). Cells from each group were washed with pre-chilled PBS and the supernatant discarded. Washed cells were lysed with an equal volume of RIPA lysate (P0013B, Beyotime) in an ice bath for 5 min and centrifuged at 14,000 rpm and 4°C for 10 min. A portion of the cell extract was removed as input, and the remainder was incubated with the antibody for co-precipitation. RNA was extracted from the samples and input after detachment with proteinase K for subsequent qRT-PCR detection of miR-499 and CK2α expression. The antibody used for RIP (rabbit anti-Ago2 (1:100, ab32381, Abcam, UK) was mixed for 30 min at room temperature, and rabbit anti-human IgG (1:100, ab109489, Abcam, UK) was used as a negative control. Each experiment was repeated three times.

Dual-luciferase reporter gene assay

As per the binding sequence between CK2α mRNA 3′ untranslated region (UTR) and miR-499, the target sequence and the mutant sequence were designed and synthesized. The artificially synthesized gene fragments of CK2α 3′ UTR and the mutant were introduced into the pGL3 vector (Promega, Madison, WI, USA) for the construction of mutant-type (MUT) plasmids. After transfection for 48 h, the luciferase reporter plasmids wild type (WT) or MUT were co-transfected with miR-499 mimic or NC mimic into HEK293T cells, which were then collected and lysed. Luciferase activity was measured using a Luminometer TD-20/20 detector (E5311, Promega, Madison, WI, USA) and Dual-Luciferase Reporter Assay System kit (Promega, Madison, WI, USA).

Plasmid transfection

Fibroblasts (4 × 10^5 cells/well) at the logarithmic growth phase were inoculated into 6-well cell plates. When cells reached 80%–90% confluence, the cells were transfected with mimic-NC (sense: 5′-UCACAAC CUCCUAGAAAGAGUAGA-3′, antisense: 5′-UCUACUCUUUCUA

GGAGGUUGUGA-3′), miR-499 mimic (sense: 5′-UUAAGACUUG CAGUGAUGUUU-3′, antisense: 5′-AAACAUCACUGCAAGUCUU AA-3′), inhibitor-NC (5′-UCACAACCUCCUAGAAAGAGUAGA -3′), miR-499 inhibitor (5′- AAACAUCACUGCAAGUCUUAA-3′), sh-NC (5′-UAGCGACUAAACACAUCAA-3′), or sh-CK2α (5′-UG UCCGAGUUGCUUCCCGA-3′) in accordance with the manufacturer's instructions using a Lipofectamine 2000 kit (11668-019, Invitrogen, Carlsbad, CA, USA). Transfection sequences and plasmids were purchased from Shanghai GenePharma (Shanghai, China).

Statistical analysis

Statistical analysis was performed using SPSS 21.0 software (IBM, Armonk, NY, USA). Following the application of a homogeneity test of normal distribution and variance, data conforming to normal distribution were expressed as the mean ± standard deviation. Data between the two groups were compared using an independent sample t test. Comparisons between multiple groups were conducted by one-way analysis of variance (ANOVA), followed by Tukey multiple comparison test. Statistical analysis in relation to time-based measurements within each group was analyzed using repeated-measures ANOVA, followed by a Bonferroni's post hoc test for multiple comparisons. Statistical analysis in relation to different concentrations within each group was analyzed using two-way ANOVA, followed by Bonferroni's post hoc test. ROC was applied to identify the optimal cutoff value for miR-499 expression, whereas the Kaplan-Meier method was employed to analyze the relationship between high and low expression of miR-499 in the lung cancer tissues and total survival (log-rank test). A p value < 0.05 was considered to be indicative of statistically significant difference.

The datasets generated and/or analyzed during the current study are available from the corresponding author on reasonable request.

SUPPLEMENTAL INFORMATION

Supplemental information can be found online at https://doi.org/10.1016/j.omto.2021.03.016.

ACKNOWLEDGMENTS

The authors would like to acknowledge the helpful suggestions concerning this study received from Dr. Qin Lin. This study was supported partly by grants from the National Natural Science Foundation of China (81472202, 81972214, 81372175, and 82071956); Shanghai Natural Science Foundation (20ZR1472400); Natural Science Foundation of Hunan Province of China (2020WK2020); The Fundamental Research Funds for the Central Universities (22120170212 and 22120170117); Program of Shanghai Academic/Technology Research Leader (18XD1403000); Construction of Clinical Medical Center for Tumor Biological Samples in Nantong (HS2016004); The Peak of Six Personnel Foundation in Jiangsu Province (WSW-009); Jiangsu 333 Program (BRA2017205); Nantong Medical Key Talents Training Plan (Key 43); Nantong Science and Technology Project (yyz15026); and Wu Jieping Medical Foundation (320.6750.14326).

AUTHOR CONTRIBUTIONS

Y.S.M., B.W.S., G.X.J., J.B.L., and D.F. designed the study. Y.S.M., H.M.L., P.F.X., R.X., Y.S., Z.J.W., F.Y., J.B.L., Y.Z.Y., L.K.H., C.Y.J., W.W., Z.W.L., and G.R.W. collated the data, carried out data analyses, and produced the initial draft of the manuscript. J.B.L., G.X.J., and D.F. contributed to drafting the manuscript. H.M.L., P.F.X., and R.X. contributed to revising the manuscript and responding to the reviewers. All authors have read and approved the final submitted manuscript.

DECLARATION OF INTERESTS

The authors declare no competing interests.

REFERENCES

1. Wang, Y.F., Ao, X., Liu, Y., Ding, D., Jiao, W.J., Yu, Z., Zhai, W.X., Dong, S.H., He, Y.Q., Guo, H., and Wang, J.X. (2019). Microrna-608 promotes apoptosis in non-small cell lung cancer cells treated with doxorubicin through the inhibition of tfap4. Front. Genet. *10*, 809.
2. González-Marrón, A., Martín-Sánchez, J.C., Miró, Q., Matilla-Santander, N., Cartanyà-Hueso, À., Mucci, L., and Martínez-Sánchez, J.M. (2019). Relation between tobacco control policies and population at high risk of lung cancer in the European Union. Environ. Res. *179* (Pt A), 108594.
3. Liu, J., Yao, L., Zhang, M., Jiang, J., Yang, M., and Wang, Y. (2019). Downregulation of LncRNA-XIST inhibited development of non-small cell lung cancer by activating miR-335/SOD2/ROS signal pathway mediated pyroptotic cell death. Aging (Albany NY) *11*, 7830–7846.
4. Khandelwal, A., Seam, R.K., Gupta, M., Rana, M.K., Prakash, H., Vasquez, K.M., and Jain, A. (2020). Circulating microRNA-590-5p functions as a liquid biopsy marker in non-small cell lung cancer. Cancer Sci. *111*, 826–839.
5. Zanoaga, O., Braicu, C., Jurj, A., Rusu, A., Buiga, R., and Berindan-Neagoe, I. (2019). Progress in research on the role of flavonoids in lung cancer. Int. J. Mol. Sci. *20*, 4291.
6. Halvorsen, A.R., Ragle Aure, M., Õjlert, A.K., Brustugun, O.T., Solberg, S., Nebdal, D., and Helland, Å. (2019). Identification of microRNAs involved in pathways which characterize the expression subtypes of NSCLC. Mol. Oncol. *13*, 2604–2615.
7. Sun, C.C., Li, S.J., Zhang, F., Zhang, Y.D., Zuo, Z.Y., Xi, Y.Y., Wang, L., and Li, D.J. (2016). The novel mir-9600 suppresses tumor progression and promotes paclitaxel sensitivity in non-small-cell lung cancer through altering stat3 expression. Mol. Ther. Nucleic Acids *5*, e387.
8. Sun, C.C., Zhu, W., Li, S.J., Hu, W., Zhang, J., Zhuo, Y., Zhang, H., Wang, J., Zhang, Y., Huang, S.X., et al. (2020). FOXC1-mediated LINC00301 facilitates tumor progression and triggers an immune-suppressing microenvironment in non-small cell lung cancer by regulating the HIF1α pathway. Genome Med. *12*, 77.
9. Wu, J., Sun, B., Zhang, S., Zhang, J., Tong, J., Nie, J., and Li, J. (2019). Effects of radon on miR-34a-induced apoptosis in human bronchial epithelial BEAS-2B cells. J. Toxicol. Environ. Health A *82*, 913–919.
10. Qiu, F., Yang, L., Ling, X., Yang, R., Yang, X., Zhang, L., Fang, W., Xie, C., Huang, D., Zhou, Y., and Lu, J. (2015). Sequence variation in mature microrna-499 confers unfavorable prognosis of lung cancer patients treated with platinum-based chemotherapy. Clin. Cancer Res. *21*, 1602–1613.
11. Li, M., Zhang, Q., Wu, L., Jia, C., Shi, F., Li, S., Peng, A., Zhang, G., Song, X., and Wang, C. (2014). Serum miR-499 as a novel diagnostic and prognostic biomarker in non-small cell lung cancer. Oncol. Rep. *31*, 1961–1967.
12. Iwama, H., Murao, K., Imachi, H., and Ishida, T. (2011). MicroRNA networks alter to conform to transcription factor networks adding redundancy and reducing the repertoire of target genes for coordinated regulation. Mol. Biol. Evol. *28*, 639–646.
13. Liu, Y., Amin, E.B., Mayo, M.W., Chudgar, N.P., Bucciarelli, P.R., Kadota, K., Adusumilli, P.S., and Jones, D.R. (2016). CK2α′ Drives Lung Cancer Metastasis by Targeting BRMS1 Nuclear Export and Degradation. Cancer Res. *76*, 2675–2686.

14. Zhang, S., Long, H., Yang, Y.L., Wang, Y., Hsieh, D., Li, W., Au, A., Stoppler, H.J., Xu, Z., Jablons, D.M., and You, L. (2013). Inhibition of CK2α down-regulates Notch1 signalling in lung cancer cells. J. Cell. Mol. Med. *17*, 854–862.

15. Zhang, S., Yang, Y.L., Wang, Y., You, B., Dai, Y., Chan, G., Hsieh, D., Kim, I.J., Fang, L.T., Au, A., et al. (2014). CK2α, over-expressed in human malignant pleural mesothelioma, regulates the Hedgehog signaling pathway in mesothelioma cells. J. Exp. Clin. Cancer Res. *33*, 93.

16. Li, L., Wu, J., Zheng, F., Tang, Q., Wu, W., and Hann, S.S. (2016). Inhibition of EZH2 via activation of SAPK/JNK and reduction of p65 and DNMT1 as a novel mechanism in inhibition of human lung cancer cells by polyphyllin I. J. Exp. Clin. Cancer Res. *35*, 112.

17. Li, W., Li, H., Zhang, L., Hu, M., Li, F., Deng, J., An, M., Wu, S., Ma, R., Lu, J., and Zhou, Y. (2017). Long non-coding RNA LINC00672 contributes to p53 protein-mediated gene suppression and promotes endometrial cancer chemosensitivity. J. Biol. Chem. *292*, 5801–5813.

18. Yang, X., Li, X., and Zhou, B. (2018). A meta-analysis of mir-499 rs3746444 polymorphism for cancer risk of different systems: Evidence from 65 case-control studies. Front. Physiol. *9*, 737.

19. Li, Q., Li, K., Yang, T., Zhang, S., Zhou, Y., Li, Z., Xiong, J., Zhou, F., Zhou, X., Liu, L., et al. (2017). Association of protein kinase CK2 inhibition with cellular radiosensitivity of non-small cell lung cancer. Sci. Rep. *7*, 16134.

20. Prensner, J.R., and Chinnaiyan, A.M. (2011). The emergence of lncRNAs in cancer biology. Cancer Discov. *1*, 391–407.

21. Li, N., Ma, J., Guarnera, M.A., Fang, H., Cai, L., and Jiang, F. (2014). Digital PCR quantification of miRNAs in sputum for diagnosis of lung cancer. J. Cancer Res. Clin. Oncol. *140*, 145–150.

22. Li, M., Zhang, S., Wu, N., Wu, L., Wang, C., and Lin, Y. (2016). Overexpression of miR-499-5p inhibits non-small cell lung cancer proliferation and metastasis by targeting VAV3. Sci. Rep. *6*, 23100.

23. Li, X., Wang, J., Jia, Z., Cui, Q., Zhang, C., Wang, W., Chen, P., Ma, K., and Zhou, C. (2013). MiR-499 regulates cell proliferation and apoptosis during late-stage cardiac differentiation via Sox6 and cyclin D1. PLoS ONE *8*, e74504.

24. Wang, B.Q., Yang, B., Yang, H.C., Wang, J.Y., Hu, S., Gao, Y.S., and Bu, X.Y. (2018). MicroRNA-499a decelerates glioma cell proliferation while accelerating apoptosis through the suppression of Notch1 and the MAPK signaling pathway. Brain Res. Bull. *142*, 96–106.

25. Zhao, S., Zhen, Y., Fu, L., Gao, F., Zhou, X., Huang, S., and Zhang, L. (2019). Design, synthesis and biological evaluation of benzamide derivatives as novel NTCP inhibitors that induce apoptosis in HepG2 cells. Bioorg. Med. Chem. Lett. *29*, 126623.

26. Shi, Y., Han, Y., Niu, L., Li, J., and Chen, Y. (2019). MiR-499 inhibited hypoxia/reoxygenation induced cardiomyocytes injury by targeting SOX6. Biotechnol. Lett. *41*, 837–847.

27. Lee, Y.H., Kim, S.Y., and Bae, Y.S. (2014). Upregulation of miR-760 and miR-186 is associated with replicative senescence in human lung fibroblast cells. Mol. Cells *37*, 620–627.

28. Liang, Y., Inhibition of MiRNA-125b Decreases Cerebral Ischemia/Reperfusion Injury by Targeting CK2α/NADPH Oxidase Signaling. Cell. Physiol. Biochem. 45, 1818–1826.

29. Cardozo, C.P. (2019). Identification of transcription factor-binding sites in the mouse foxo1 promoter. Methods Mol. Biol. *1890*, 29–40.

30. Ni, Z., He, J., Wu, Y., Hu, C., Dai, X., Yan, X., Li, B., Li, X., Xiong, H., Li, Y., et al. (2018). AKT-mediated phosphorylation of ATG4B impairs mitochondrial activity and enhances the Warburg effect in hepatocellular carcinoma cells. Autophagy *14*, 685–701.

31. Trembley, J.H., Kren, B.T., Abedin, M.J., Shaughnessy, D.P., Li, Y., Dehm, S.M., and Ahmed, K. (2019). CK2 Pro-Survival Role in Prostate Cancer Is Mediated via Maintenance and Promotion of Androgen Receptor and NFκB p65 Expression. Pharmaceuticals (Basel) *12*, 89.

32. Yin, M., Ren, X., Zhang, X., Luo, Y., Wang, G., Huang, K., Feng, S., Bao, X., Huang, K., He, X., et al. (2015). Selective killing of lung cancer cells by miRNA-506 molecule through inhibiting NF-κB p65 to evoke reactive oxygen species generation and p53 activation. Oncogene *34*, 691–703.

33. Chen, Y., Tang, Q., Wu, J., Zheng, F., Yang, L., and Hann, S.S. (2015). Inactivation of PI3-K/Akt and reduction of SP1 and p65 expression increase the effect of solamargine on suppressing EP4 expression in human lung cancer cells. J. Exp. Clin. Cancer Res. *34*, 154.

OMTO, Volume *21*

Supplemental information

MicroRNA-499 serves as a sensitizer for lung cancer cells to radiotherapy by inhibition of CK2α-mediated phosphorylation of p65

Yu-Shui Ma, Bo-Wen Shi, Hai-Min Lu, Peng-Fei Xie, Rui Xin, Zhi-Jun Wu, Yi Shi, Yu-Zhen Yin, Li-Kun Hou, Cheng-You Jia, Wei Wu, Zhong-Wei Lv, Fei Yu, Gao-Ren Wang, Ji-Bin Liu, Geng-Xi Jiang, and Da Fu

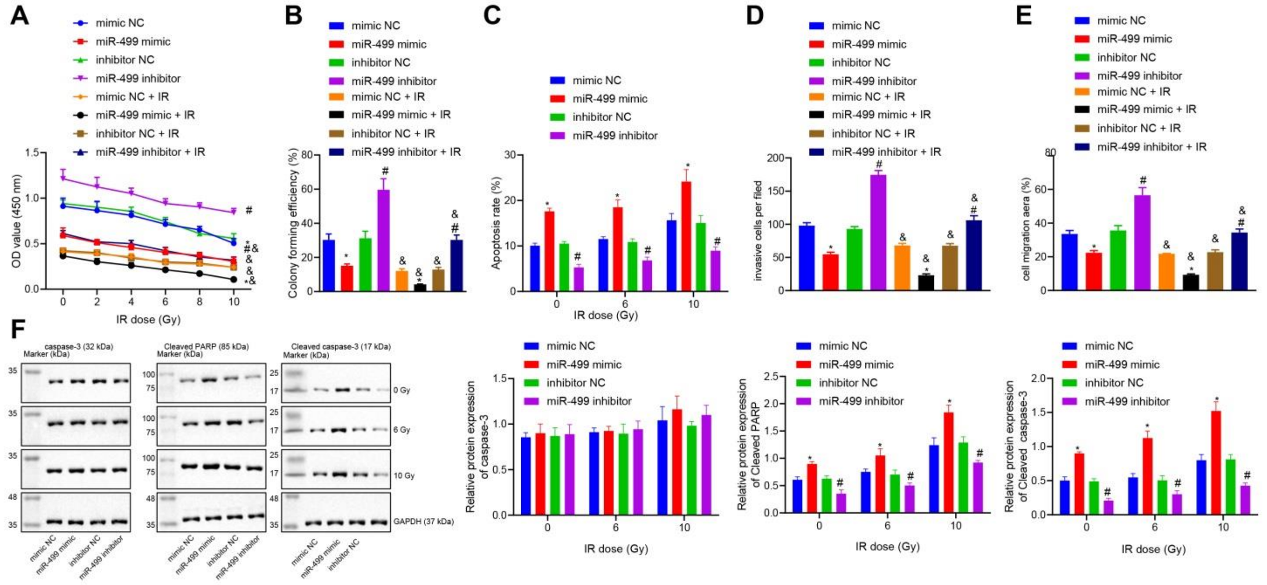

Supplementary Figure 1. Overexpression of miR-499 increased the sensitivity of lung cancer NCI-H292 cells to irradiation (IR) and promoted IR-induced apoptosis *in vitro.* A, Sensitivity of lung cancer cells NCI-H292 to IR detected by MTT assay after overexpression or silencing of miR-499. B, Colonies as measured by colony formation assay in NCI-H292 cells overexpressing or silencing miR-499. * $p < 0.05$ *vs.* NCI-H292 cells treated with mimic NC; # $p < 0.05$ *vs.* NCI-H292 cells transfected with inhibitor NC. C, Apoptosis of NCI-H292 cells as evaluated by flow cytometry after overexpression or silencing of miR-499. D, Invasion assay was performed to examine the effect of overexpression or interference of miR-499 on irradiation-induced invasion of lung cancer cells NCI-H292. E, Scratch assay was performed to examine the effect of miR-499 mimic or miR-499 inhibitor on the migration ability of lung cancer cells NCI-H292 induced by irradiation. F, The protein expression of Caspase-3, Cleaved PARP and Cleaved Caspase-3 measured by Western blot analysis in NCI-H292 cells overexpressing or silencing miR-499. * $p < 0.05$ *vs.* NCI-H292 cells treated with mimic NC; # $p < 0.05$ *vs.* NCI-H292 cells transfected with inhibitor NC. & $p < 0.05$ *vs.* the group with no irradiation. n = 3. The measurement data were expressed as mean ± standard deviation. Data between two groups were compared using independent sample *t*-test. Comparisons among multiple groups were conducted by ANOVA, followed by Bonferroni's *post-hoc* test.

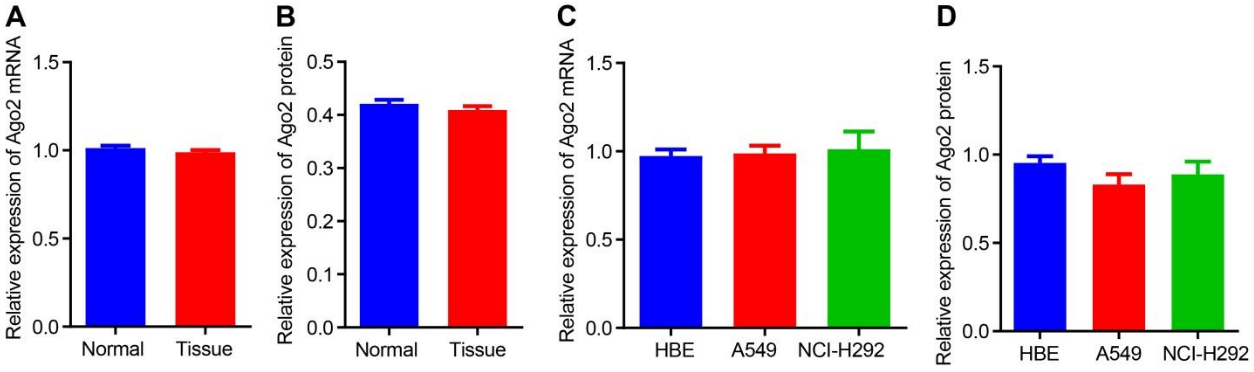

Supplementary Figure 2. A, RT-qPCR was used to detect the difference of Ago2 expression levels between cancer tissues and adjacent normal tissues. B, Western blot was used to detect the difference of Ago2 expression levels between cancer tissues and adjacent normal tissues. C, RT-qPCR was used to detect the difference of Ago2 expression levels between lung cancer cells and immortalized HBE cells. D, Western blot was used to detect the difference of Ago2 expression levels between lung cancer cells and immortalized HBE cells.

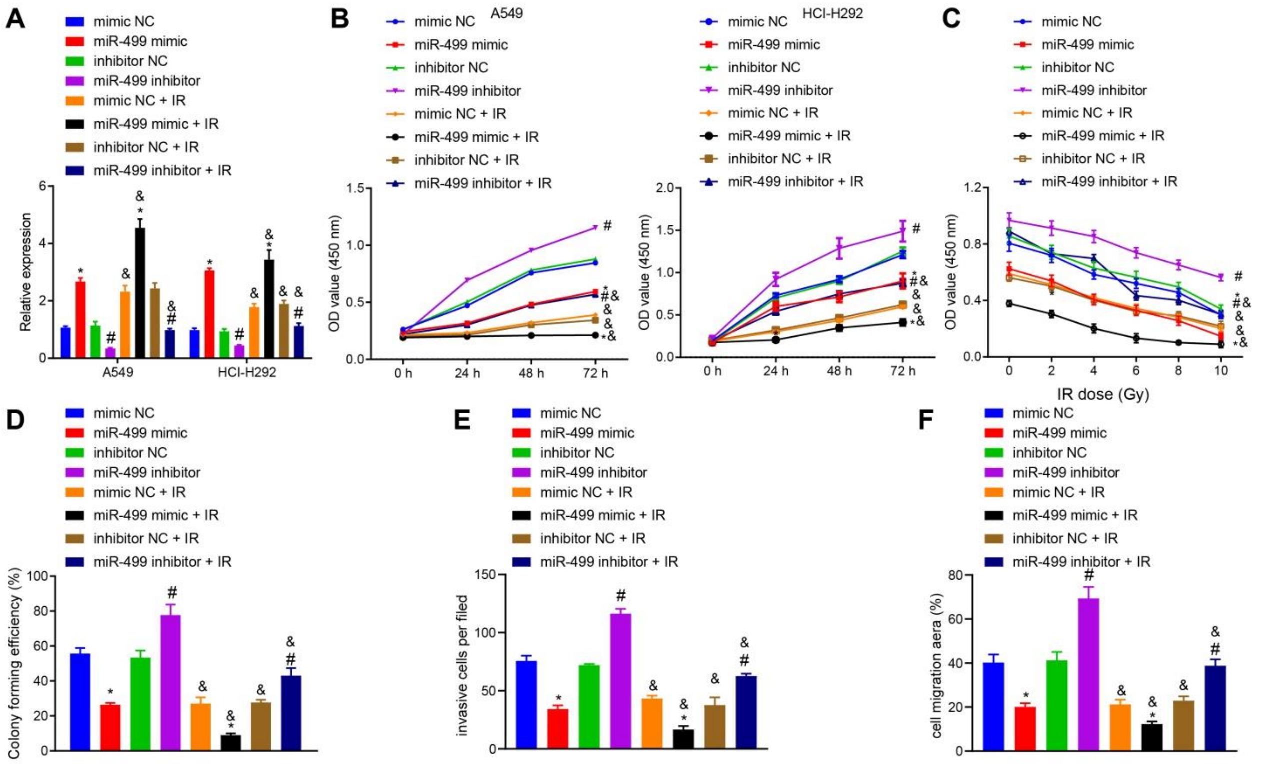

Supplementary Figure 3. A, Western blot was employed to verify the regulatory relationship between CK2α and p65 phosphorylation in A549 cells. B, After 10 Gy irradiation, the effect of miR-499 in A549 cells on radiosensitivity by targeting CK2α was verified by colony formation assay. C, MTT assay was used to detect the sensitivity of lung cancer cells to irradiation. * $p < 0.05$ compared with the miR-499 inhibitor group, and # $p < 0.05$ compared with the inhibitor NC + CX-4945 group. D, Western blot was used to detect the effect of miR-499 on regulating p65, p-p65/p65, Cleaved PARP, caspase-3, and Cleaved caspase-3 expression by targeting CK2α in A549 cells, * $p < 0.05$ compared with the miR-499 inhibitor group and # $p < 0.05$ compared with the inhibitor NC + CX-4945 group. Data were expressed as mean ± standard deviation, independent samples *t*-test was used for comparison between two groups.

Table S2 Primer sequences for RT-qPCR

Gene	Primer sequence (5'-3')	
miR-499	F: TTAAGACTTGCAGTGATGTTT	R: AAACATCACTGCAAGTCTTAA
CK2α	F: TGTTCGTCATGGGTGTGAAC	R: TGGACTGCTTGTGGCTGTGG
GAPDH	F: CCCACTCCTCCACCTTTGAC	R: CATACCAGGAAATGAGCTTGACAA
U6	F: CGACAAGACGATCCGGGTAAA	R: GGTTGAGGAGTGGGTCGAAG

Note: RT-qPCR, reverse transcription quantitative polymerase chain reaction; CK2α, casein kinase 2 alpha; GAPDH, glyceraldehyde-3-phosphate dehydrogenase; F, forward; R, reverse.
